# The Nursing Profession: Reconstruction v. Destruction

**Published:** 1919-04-05

**Authors:** Gladys M. E. Leigh


*To the Editor of* The Hospital.

### The Nursing Profession: Reconstruction v. Destruction.

*To the Editor of* The Hospital.

Sir,—Your correspondent "Ierne" places herself in the position of an owner of property who would destroy our habitation before the plans for rebuilding are matured. She admits she is dealing with probabilities, not facts, nor is "Ierne" just in her attitude towards the training schools. One of the London training schools—second to none in Great Britain and Ireland—admits probationer nurses at the age of twenty-one. Many of the provincial schools do likewise. Also "Ierne" infers that the science of nursing will not keep pace automatically with the science of medicine unless probationers are converted into students. Never was there a greater fallacy or a greater reflection upon the *esprit de corps* of the nursing services. Reconstruction cannot take place unless there is an organised body with authority to act, possessing the confidence of the rank and file. Let the College of Nursing prove itself to be this living virile organisation with the good of all grades of the nursing service at heart, that it stands impartially to uphold the best traditions of the nursing services, to protect the interests of the employers and to safeguard the employees by insisting upon a shorter working day and a living wage. Let the College maintain the balance with a steady hand and seek to reconcile the extremists.

The first step towards reconstruction is to raise the educational status, and every thinking human being will agree with "Ierne" when she asks for shorter hours, increased pay, outside interests and an assured future. But "Ierne" fails to realise that hospital training is unique, although the nurse herself is no different from other trained workers. Has "Ierne" considered that the training of a medical student is considerably longer than that of a nurse probationer; also, has she inquired how many students are admitted into the wards of a hospital at the age of eighteen? If "Ierne" has faith

### THE EDITOR'S LETTER-BOX—(*continued*).

in her scheme, let her draw out a working table and submit it for analysis. If it will bear this test let a referendum be taken of the opinion of all women, trained and training, as to whether they approve of so drastic a change. Surely the opinion of the workers would carry weight. In the meantime, whilst grasping at the shadow, do not let us lose our hold of the substance.—Yours faithfully,

Gladys M. E. Leigh.

